# Optical regulation of cell chain

**DOI:** 10.1038/srep11578

**Published:** 2015-06-22

**Authors:** Xiaoshuai Liu, Jianbin Huang, Yao Zhang, Baojun Li

**Affiliations:** 1 State Key Laboratory of Optoelectronic Materials and Technologies, School of Physics and Engineering, Sun Yat-Sen University, Guangzhou 510275, China

## Abstract

Formation of cell chains is a straightforward and efficient method to study the cell interaction. By regulating the contact sequence and interaction distance, the influence of different extracellular cues on the cell interaction can be investigated. However, it faces great challenges in stable retaining and precise regulation of cell chain, especially in cell culture with relatively low cell concentration. Here we demonstrated an optical method to realize the precise regulation of cell chain, including removing or adding a single cell, adjusting interaction distance, and changing cell contact sequence. After injecting a 980-nm wavelength laser beam into a tapered optical fiber probe (FP), a cell chain of *Escherichia colis* (*E. colis*) is formed under the optical gradient force. By manipulating another FP close to the cell chain, a targeted *E. coli* cell can be trapped by the FP and removed from the chain. Further, the targeted cell can be added back to the chain at different positions to change the cell contact sequence. The experiments were interpreted by numerical simulations and the impact of cell sizes and shapes on this method was analyzed.

Increasing attention has been paid to the interaction between neighboring cells because of its crucial role in ruling cell fate and function, such as cell growth^1^,^2^,^3^, differentiation^4^,^5^,^6^, intercellular signaling pathway^7^,^8^,^9^ and mechanical force transduction^10^,^11^. The cell interaction occurs mainly through exchange of soluble signaling molecules and the direct cell–cell contact, which can vary in both time and space continuously^12^,^13^. Thus, it is of great importance in dynamically adjusting cell–cell interaction distance and contact sequence, and further individually investigating the spatiotemporal aspects of different extracellular cues. Recent advances in micropatterening^14^,^15^ and microelectromechanical systems^16^,^17^ show the invaluable performance in multi-cellular arrangements and dynamic control of tissue organization. However, the cells must be confined on an elaborated substrate which was usually not reusable in these techniques.

Recent studies have demonstrated the remarkable ability to form cell patterns by using holographic optical tweezers (HOTs)^18^,^19^,^20^,^21^, with significant advantages in achieving the formation of arbitrary patterns. The cells can also be removed from or added into the pattern by controlling the laser beam movements. However, for HOTs, appropriate algorithms are required for precise phase hologram calculation to realize a pattern of cells. The case becomes more complicated when manipulating targeted cells from a pattern because the movements of the laser beams for manipulation and the retaining of the other beams are both required. In addition, HOTs were usually built on an optical system consisted of dichroic mirrors, spatial light modulators and high-numerical-aperture focusing objectives. The working distance of the objectives limits the depth at which cells can be manipulated in the cell suspensions. Besides HOTs, surface plasmon-based optical tweezers (SPOTs)^22^,^23^ have been demonstrated to form cell patterns with high trapping stability and retaining ability, especially for the sub-micro sized cells and biological molecules. However, for SPOTs, the specific patterns of cells are decided by the carefully designed lithographic substrates, making it difficult to dynamically adjust the cell–cell contact. Moreover, in SPOTs approaches, the formation of cell patterns is also limited at a specific depth of the cell suspension. Notably, formation of cell chain is an alternative approach to realize the cell–cell contact. A very simple and efficient way to form a cell chain, especially in the cell culture with relatively low concentration, is to use an optical fiber probe (FP)^24^. The method is free from the limitation on the depth of cell manipulation in the suspensions and does not require any elaborated substrates. Unfortunately, the cell contact sequence and interaction distance are fixed once the cell chain is formed, which limit the further investigation of extracellular cues on the interaction. Therefore, in this work, we present an optical method to realize the precise regulation of cell chain, including changing cell contact sequence and adjusting interaction distance. A tapered FP was used for the formation of *Escherichia coli* (*E. coli*) cell chain to realize the cell–cell contact. By using another taper FP, a targeted cell can be removed from the cell chain and then added back into it, realizing the regulation of cell chain.

## Results

### Schematic for the regulation of cell chain

**Figure 1:**
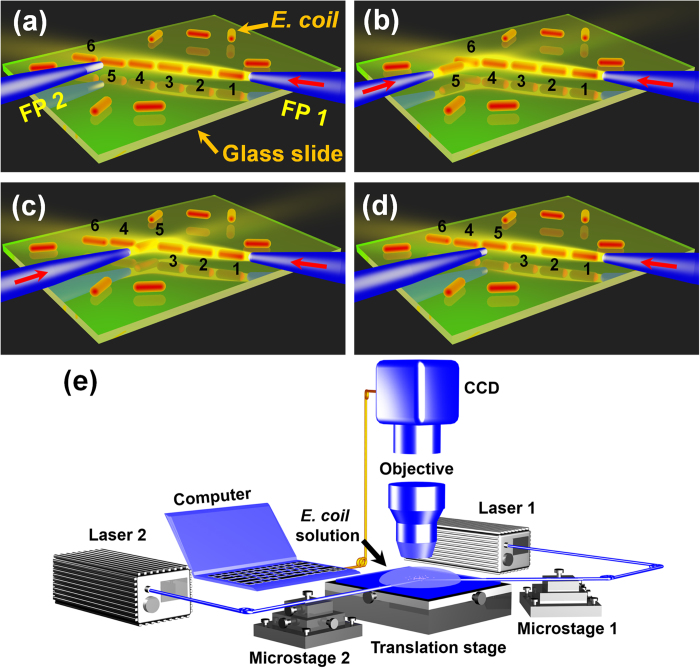
Schematic of the regulation process and experimental setup. (**a**) A cell chain is formed after a laser beam is injected into FP 1. (**b**) With a laser beam injected into FP 2, *E. coli* 5 is rotated and then removed from the cell chain. (**c**) *E. coli* 5 is added back at a new position into chain. (**d**) After turning off the laser in FP 2, *E. coli* 5 is orientated along the axial direction of FP 1. (**e**) Schematic of the experiment setup.

Figure 1 schematically shows the formation and regulation process of an *E. coli* cell chain. Once a laser beam is injected into FP 1, *E. colis* are trapped one after another along the axial direction of FP 1 to form a chain under the optical gradient force (Fig. 1a)^24^. For illustration, a cell chain consisted of six trapped *E. colis* is shown in Fig. 1. Then FP 2 is adjusted to approach *E. coli* 5. After a laser beam is injected into FP 2, *E. coli* 5 is rotated and orientated toward the axial direction of FP 2. FP 2 is shifted until *E. coli* 5 is removed from the chain so that the cell contact sequence is changed from 1–2–3–4–5–6 to 1–2–3–4–6 (Fig. 1b). Then *E. coli* 5 is put between the *E. coli* 3 and 4 by moving FP 2 (Fig. 1c). After turning off the laser in FP 2, *E. coli* 5 is gradually rotated and orientated along the axial direction of FP 1 (Fig. 1d), changing the cell contact sequence again (from 1–2–3–4–6 to 1–2–3–5–4–6). Figure 1e schematically shows the experiment setup. FPs 1 and 2 (see methods for fabrication) were connected to the lasers 1 and 2, respectively. The wavelength of the both lasers is 980 nm, which is weakly absorbed by most living matter^25^. More importantly, 980 nm has been shown to be a good wavelength to prevent optical damage in bacteria and mammalian cells^26^. After sheathed with a glass capillary, FPs 1 and 2 were fixed on microstages 1 and 2, respectively. The probe tips were immersed in the *E. coli* solution (see methods for the suspension preparation) which was dropped on a glass slide with an injector. The slide was mounted on an *x*–*y* manual translation stage (resolution: 50 nm) to achieve fine positioning and mechanical stability. An optical microscope incorporated with a charged coupled device (CCD) was used for the real time monitoring, image capturing and video recording.

### Design and numerical calculation

**Figure 2:**
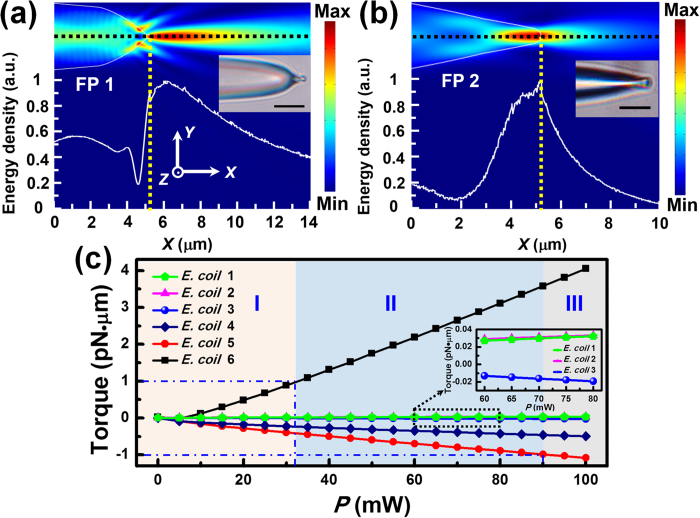
Simulated energy density distribution and calculated optical torque. (**a,b**) Simulated energy density distributions of FPs 1 and 2 with normalized energy densities along the fiber axial direction. The yellow dashed lines show the positions of fiber tips. The insets are the microscopic images of the FPs. Scale bar: 5 μm. (**c**) Calculated torques exerted on the 6 *E. colis* as a function of the power in FP 2 (power in FP 1: 30 mW). The inset shows the torques on the *E. coils* 1, 2 and 3 with the power ranged from 60 to 80 mW to distinguish the curves for the three cells.

As shown in Fig. 1, the functions of FPs 1 and 2 are forming the *E. coli* chain and manipulating a targeted cell, respectively. Since the optical field distributions outputted from the FPs are strongly affected by the fiber tips, a specific shape is required for each of the two FPs. The shapes were designed based on a series of field distribution simulations for different FPs. The simulations were performed by a two-dimensional finite element method (COMSOL Multiphysics 4.3). Distributions of the electric field ***E***, magnetic field ***H*** and the electromagnetic energy density *w*, expressed by *w* = (***E*****•*****D*** + ***H*****•*****B***)/2 where ***D*** is the electric displacement vector and ***B*** is the magnetic flux density vector, were obtained by simulations. Figure 2a, b show the energy density distributions of FPs 1 and 2 with proper shapes, respectively. The refractive indices were set to be 1.44, 1.33 and 1.39 for the fiber, water and *E. coli*, respectively^27^. The *E. coli* in the simulations was assumed to be a homogeneous rod (length *L*: 1.5 μm; diameter *D*: 500 nm) with hemispherical caps. The curves in Fig. 2a, b show the normalized energy density along the fiber axial direction as indicated by the black dashed lines. The laser intensity outputted from the FPs varies with the distance to the fiber tips. It can be seen that for FPs 1 and 2, the decay speeds of the intensity with the distance are different due to the different tip shapes (Fig. 2a,b). For FP 2, the normalized energy density (along the fiber axial direction) decreased from 1 to 0.4 within 1 μm. It is indicated that FP 2, with a much more concentrated energy density at the fiber tip, is suitable for manipulating a single cell. While for FP 1, the density decreased from 1 to 0.4 within 8 μm, indicating that the outputted light from the tip of FP 1 decreases much more slowly and covers a much longer distance. Therefore, FP 1 can be used for forming the cell chain^24^. It should be noted the cell number in the cell chain increased with the power of FP 1. Moreover, the cell number also depended on the local cell concentration around the tip of FP 1.

Besides, the angle between FPs (*θ*) should be set a proper value to realize the optimal regulation of cell chain. According to the experiment results and theoretical simulation, it can be known that for *θ *< 30°, the taken cell by FP 2 will be easily trapped by FP 1 again, which decreases the orientation stability. While for *θ* > 60°, the power in FP 2 will be larger to remove the specific cell from the cell chain, which increases the photo-toxicity for cells. Thus, *θ* should be ranged from 30° to 60°. During the experiment, it was set to be 50°. Further, the laser power injected into FP 1 was fixed at 30 mW to retain the formation of *E. coli* chain. To realize single cell manipulation without affecting other *E. colis* in the chain, a proper value is required for the power in FP 2 (*P*). Thus, optical torques (*T*) exerted on the six *E. colis* in the chain were calculated as a function of *P* (Fig. 2c). The optical torque *T* is defined as^28^:


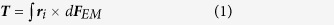


where ***r***_*i*_ is the position vector pointing from the central point of *E. coli* to the interaction point, *d**F***_*EM*_ is the electromagnetic force element exerted on the interaction point *i*. ***F***_*EM*_ can be expressed as^29^:


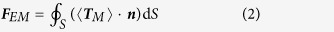


where *dS* is the surface element surrounding the cell, ***n*** is the surface normal vector, 〈***T***_*M*_〉 is the time-independent Maxwell stress tensor which can be derived from the simulations. Note that since the Maxwell stress tensor was used in the calculations, the obtained optical forces and torques are related to all the interaction of light with matter, *i.e*., the calculated torques were contributed by both the scattering torque and absorption torque. It should be pointed out that the absorption torque is very small, which is because of the weak absorption (*α *≈ 0.5 cm^−1^) of biological cells for the 980 nm laser^30^. According to our calculation, the absorption torque is two orders of magnitudes smaller than the resultant torque. The optical torque along +***z*** direction is defined as positive, under which the cell will be rotated counterclockwise. From Fig. 2c, it can be seen the absolute values of the torques |*T*| on the *E. colis* increase linearly as *P* increases. In the region I, as |*T*| is less than 1 pN·μm, the *E. colis* remain oriented along the axial direction of FP 1. At *P* = 32 mW, the torque exerted on *E. coli* 6 reaches 1 pN·μm and the *E. coli* starts to be rotated counterclockwise toward FP 2 (region II). After *P* increased to 90 mW, the torque exerted on *E. coli* 5 also reaches 1 pN·μm so that *E. coli* 5 will also be rotated and trapped by FP 2 (region III).

Meanwhile, the biological safety of this method should be also assayed since it is developed for biological study. To study the cell viability, 5% Trypan blue was added into the cell suspension during the regulation progress. For alive cell, it is able to prevent the dye from entering the cytoplasm due to the permselectivity mechanism of cell membrane. While for the dead one, the dye will penetrate the cell membrane and the whole cell appears blue. The results showed that for *P* < 100 mW, no staining was observed which indicated the cells survived without any detectable damages. But if *P* > 100 mW, the cells will be gradually stained to be blue, which indicated the cells suffered from the photo-toxicity and cannot maintain their normal function. Therefore, to ensure cell viability and a single-cell manipulation, *P* should be ranged from 32 to 90 mW. During the regulation progress, it was set to be 50 mW.

### Trapping and removing a targeted cell

**Figure 3:**
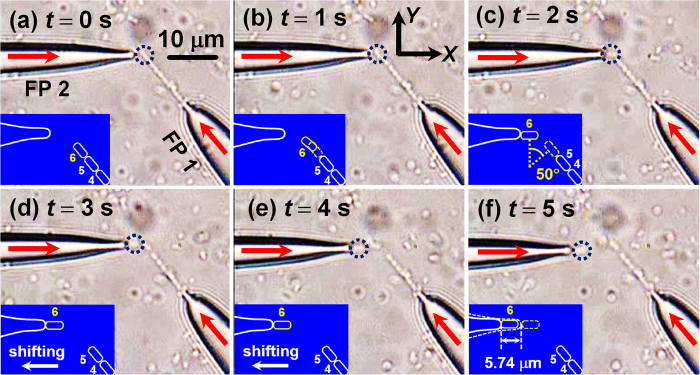
Optical microscopic images for removing *E. coli* 6 from the cell chain. (**a–c**) After the laser was injected into FP 2, *E. coli* 6 was rotated and gradually orientated along the axial direction of FP 2. (**d–f**) *E. coli* 6 was removed from the cell chain and then shifted with FP 2. The insets schematically show the removal process.

**Figure 4:**
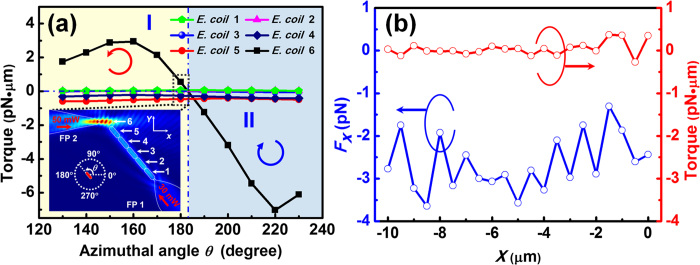
Calculated optical torque and force during the removal and shifting progress. (**a**) Calculated optical torque exerted on the six *E. colis* of the chain as a function of azimuthal angle. The inset shows the simulated energy density distribution at *θ* = 180°. The arrows indicate the rotation direction. (**b**) Calculated optical force *F*_*x*_ and torque on *E. coli* 6 with the shift of FP 2 along the −*x* direction.

After forming a cell chain consisted of 6 *E. colis* by using FP 1, the regulation operation is started by injecting the 980 nm laser (*P* = 50 mW) into FP 2 at *t* = 0 s (Fig. 3a). As FP 2 approached the end of cell chain, *E. coli* 6 started to be counterclockwise rotated and gradually orientated along the axial direction of FP 2 with a rotation angle of 50° (Fig. 3b,c). With the shift of FP 2 along the −*x* direction (Fig. 3d,e), the distance between *E. coli* 6 and *E. coli* 5 was dynamically enlarged until *E. coli* 6 was removed from the original cell chain, with a shifting distance of 5.74 μm (Fig. 3f). The detailed trapping and removal progress is shown by Movie S1 in the Supporting Information. To numerically interpret the above process, the optical field distribution was simulated at different azimuthal angles (*θ*) of *E. coli* 6 during the rotation process. Meanwhile, the optical torques exerted on the 6 *E. colis* of the chain were calculated as shown in Fig. 4a. The inset shows the energy density distribution at *θ* = 180°. In the region I (130° < *θ* < 183°), the torque exerted on *E. coli* 6 was positive so that *E. coli* 6 will be rotated counterclockwise (as indicated by the red arrow). In the region II (183° < *θ* < 230°), the torque of *E. coli* 6 was negative so that *E. coli* 6 will be rotated clockwise (as the blue arrow shown). At *θ* = 183°, the torque on *E. coli* 6 was zero and so *E. coli* 6 will be stably trapped and oriented along the axial direction of FP 2. From Fig. 4a, it is also indicated that during the rotation process of *E. coli* 6, the torques exerted on the other *E. colis* of the chain were nearly zero so that they kept trapped by FP 1. In addition, the torque and *F*_*x*_ (the *x* component of *F*_*EM*_) exerted on *E. coli* 6 were calculated with the shift of FP 2 along the −*x* direction, as shown in Fig. 4b. To indicate the direction of *F*_*x*_, +*x* direction is defined as positive. It can be seen that the torque was nearly zero and *F*_*x*_ was negative (i.e., toward the FP 2) during the shift of FP 2. Therefore, *E. coli* 6 kept trapped by FP 2 and was shifted with FP 2. Note that *E. coli* 5 was also rotated a bit during the shift of FP 2 (Fig. 3e). This was because a slight shift of FP 2 also occurred along −*y* direction in the experiment, increasing the positive torque exerted on *E. coli* 5. In addition, although Fig. 3 shows the removal of the *E. coli* at the end of the cell chain, a targeted *E. coli* at any other location of the chain can be trapped and removed from the chain with this method. For example, experiment was also performed for removing *E. coli* 2 in the chain of nine cells using FP 2 (see Fig. S1 in the Supporting Information).

### Adding the cell back into cell chain

**Figure 5:**
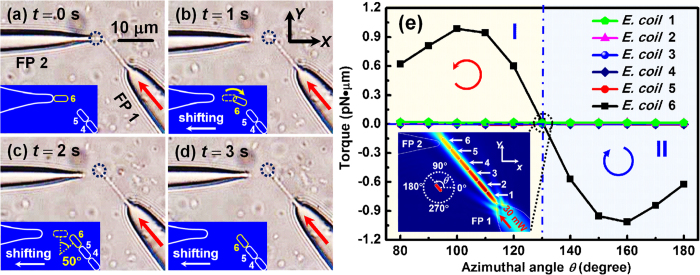
Optical microscopic images and calculated optical torque for adding *E. coli* 6 back into the cell chain. (**a–c**) After the laser in FP 2 was turned off, *E. coli* 6 was rotated and gradually orientated along the axial direction of FP 1. The insets schematically show the adding process. (**d**) As FP 2 shifted, the *E. coli* kept trapped by FP 1. (**e**) Calculated optical torque exerted on the six *E. colis* at different azimuthal angles (*θ*) of *E. coli* 6 during the rotation process. The inset shows the simulated energy density distribution at *θ* = 130°.

To further regulate the cell chain, *E. coli* 6 was added back into the original chain, as shown in Fig. 5. At *t* = 0 s, the laser in FP 2 was turned off (Fig. 5a). *E. coli* 6 was gradually rotated clockwise and finally orientated along the axial direction of FP 1 (Fig. 5b–d). The detailed adding progress is presented by Movie S2 in the Supporting Information. The torques exerted on the six *E. colis* were also calculated at different azimuthal angles (*θ*) of *E. coli* 6 during the rotation process (Fig. 5e). It can be seen that *E. coli* 6 will be rotated counterclockwise and clockwise in the region I (80° < *θ* < 130°) and region II (130° < *θ* < 180°), respectively. At *θ* = 130°, the torque on *E. coli* 6 was zero so that the *E. coli* will be stably trapped and oriented along the direction of *θ* = 130°, *i.e*., the axial direction of FP 1.

### Changing the cell contact sequence

**Figure 6:**
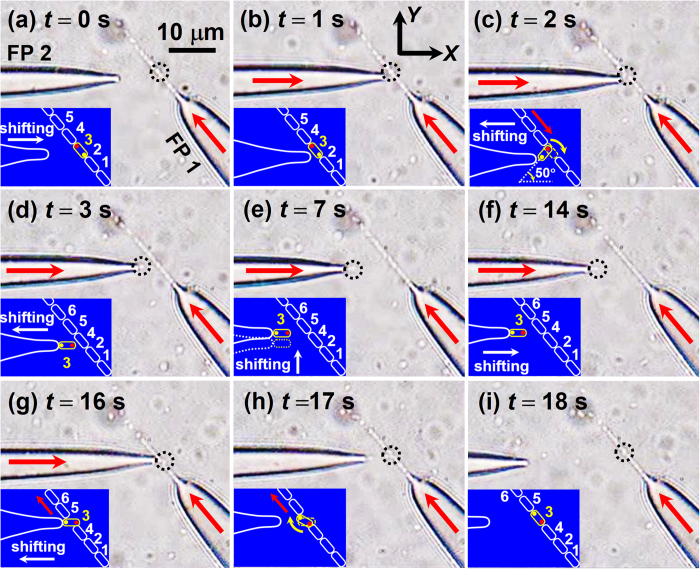
Optical microscopic images for adjusting the cell contact sequence. (**a**) FP 2 was adjusted with the microstage 1 to approach *E. coli* 3. (**b**–**d**) With the shift of FP 2 along the −*x* direction, *E. coli* 3 was rotated and then trapped by FP 2. (**e,f**) *E. coli* 3 kept trapped and shifted with FP 2 along the +*y* and +*x* direction. (**g–i**) After turning off laser in FP 2, *E. coli* 3 was added back into the cell chain between *E. coils* 4 and 5 and the contact sequence was changed to 1–2–4–3–5–6–7–8–9–10. The two tips of *E. coil* 3 were indicated by yellow and red dots.

**Figure 7:**
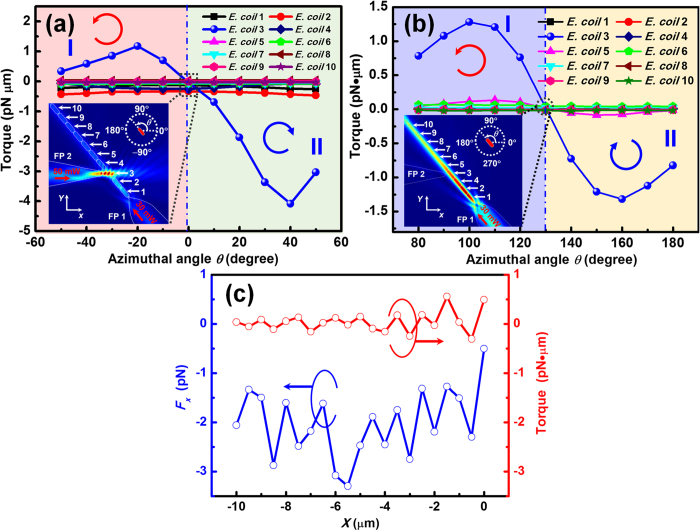
Calculated optical torque and force during the sequence regulation progress. (**a**) Calculated optical torque exerted on the 10 *E. colis* as a function of azimuthal angle *θ* (FP 1: 30 mW, FP 2: 50 mW). The inset shows the energy density distribution at *θ* = 0°. (**b**) Calculated optical torque exerted on the 10 *E. colis* as a function of azimuthal angle *θ* (FP 1: 30 mW, FP 2: 0 mW). The inset shows the energy density distribution at *θ* = 130°. (**c**) Calculated optical force *F*_*x*_ and torque on the *E. coli* 3 as a function of shifting distance.

Besides the removal and adding operations, cell contact sequence of the chain can also be regulated with this method. For illustration, cell contact sequence of a chain consisted of 10 *E. colis* was changed as shown in Fig. 6. As the above mentioned, although the laser power injected into FP 1 was fixed at 30 mW, the cell number of the chain varied due to the local cell concentration around the tip of FP 1. At *t* = 0 s, FP 2 was adjusted to approach the downside tip of *E. coli* 3 in the cell chain (Fig .6a). After turning on the laser in FP 2 (*P*_2_ = 50 mW) at *t* = 1 s (Fig. 6b), the downside tip (indicated by the yellow dot) of *E. coli* 3 was trapped by FP 2 and then shifted along −*x* direction with the shift of FP 2, while the upside tip (indicated by the red dot) remained stationary. With a shift of 1.9 μm along the −*x* direction, the angle between the axial direction of *E. coil* 3 and the +*x* direction became 50° (Fig. 6c). In this process, *E. coil* 3 was rotated clockwise and gradually orientated along the axial direction of FP 2 (Fig. 6d). Then it was removed from the cell chain and shifted along −*x* direction with a distance of 7.1 μm. To adjust the contact sequence of cell chain, FP 2 was shifted along +*y* direction with a distance of ~1 μm (Fig. 6e), followed by a shift of 9 μm along the +*x* direction (Fig. 6f). After that, *E. coli* 3 was added back into the chain between *E. coils* 4 and 5 (Fig. 6g). By turning off the laser in FP 2, *E. coli* 3 was gradually orientated along the axial direction of FP 1 so that the contact sequence of cell chain was changed to 1–2–4–3–5–6–7–8–9–10 (Fig. 6h,i). The detailed adjusting process is presented by Movie S3 in the Supporting Information. The optical torques on the *E. colis* were also calculated in the adjusting process, as shown in Fig. 7a. The torques are positive and negative in the regions I (−50° < *θ *< 0°) and II (0° < *θ *< 50°) and *E. coli* 3 will be rotated counterclockwise and clockwise (as indicated by the red and blue arrows), respectively. Therefore, *E. coli* 3 will be stably trapped and oriented along the direction at *θ* = 0°, *i.e*., the axial direction of FP 2. The torques exerted on *E. colis* after turning off the laser in FP 2 were calculated as shown in Fig. 7b. The torques were positive and negative in the region I (80° < *θ* < 130°) and II (130° < *θ* < 180°), respectively. At *θ* = 130°, the torque exerted on *E. coli* 3 was zero so that *E. coli* 3 will be stably trapped and oriented along the axial direction of FP 1. Moreover, the trapping force and torque exerted on *E. coli* 3 were calculated during the shift process of FP 2, as shown in Fig. 7c. The trapping force was negative, *i.e*. toward the FP 2, and the torque kept nearly zero during the shift process, indicating *E. coli* 3 will be stably trapped and shifted with FP 2. Note that in the operation process presented above, the total rotation angle of *E. coil* 3 was 180° rather than 0°, *i.e*., the two tips of cell (indicated by yellow and red dots in Fig. 6) was inversed in the chain. Therefore, the contact tips of two neighboring cells can also be regulated by rotating a single cell in the chain with this method. For example, by rotating *E. coli* 3 with an angle of 180° using FP 2, the *E. coli* was inversed so that the tips of *E. coil* 3 contacted with *E. colis* 2 and 4 were exchanged. (see Fig. S2 in the Supporting Information).

## Discussion

**Figure 8:**
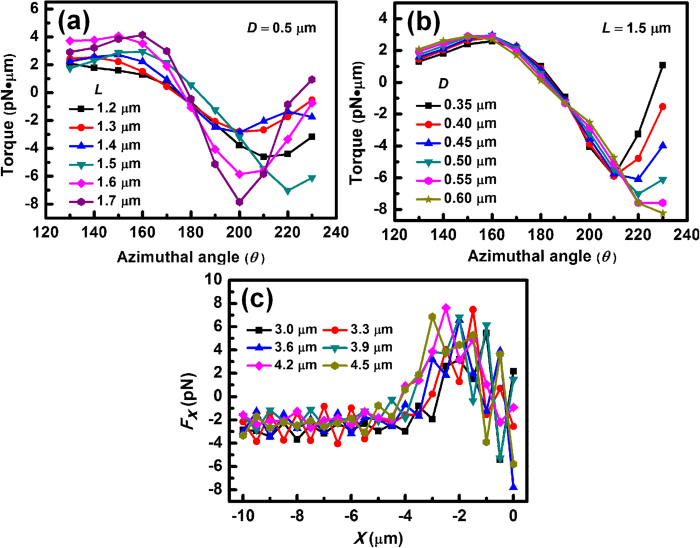
Impact of cell sizes and shapes on regulation. (**a**) Calculated optical torques on *E. colis* with lengths varied from 1.2 to 1.7 μm at different azimuthal angles. (**b**) Calculated optical torques on *E. colis* with diameters varied from 0.35 to 0.60 μm at different azimuthal angles. (**c**) Calculated trapping forces *F*_*x*_ on the targeted yeast cells with diameters varied from 3.0 μm to 4.5 μm as functions of the shift distance of FP2.

The experimental results and numerical analysis above have indicated that the contact sequence of the cell chain and the interaction distance between cells can be efficiently regulated by using two fiber probes. To further investigate the impact of cell sizes and shapes on this method, a series of simulations and calculations was performed. Figure 8a shows the optical torque exerted on *E. coli* 6 at different azimuthal angles (*θ*) when the *E. coli* is rotated and trapped by FP 2. The cell length varies from 1.2 to 1.7 μm while the diameter *D* of the cell is fixed at *D* = 0.5 μm. It can be seen the curves of the torques exerted on *E. coli* 6 with different lengths exhibit similar trends and shapes, which indicates that the presented method is valid for *E. colis* with different lengths. Moreover, at a specific azimuthal angle, the absolute value of the torque exerted on *E. coli* 6 becomes larger for longer cells. This is because the optical field at the end of the cell chain consisted of longer *E. colis* is weaker than that of a shorter chain, which leads to a smaller resisting torque (yielded by FP 1) and consequently a larger resultant torque exerted on *E. coli* 6. The torques were also calculated for the *E. colis* with different diameters (Fig. 8b). It can be seen the curves for different *E. coli* diameters also exhibit similar trends and shapes, indicating the validity of this method for *E. colis* with different diameters. Note that for the thinner cell, *e.g. D* = 0.35 μm, the resultant torque also becomes zero at *θ* = 225°. However, the *E. coli* cannot be stably oriented at *θ* = 225° because the *E. coli* at this angle is in a metastable state which will be disrupted by a perturbation. Finally, to investigate applicability of the method for cells in other shapes (*e.g*. spherical), the regulation of a yeast cell chain was also simulated and numerically analyzed. The simulated process is removing a targeted yeast cell (at the end of the chain) from a chain consisted of six yeast cells. In the simulations, the refractive index of yeast cells was set to be 1.40^31^. Note that as the yeast cells are in spherical shapes, rotation cannot be observed in any regulation process. Figure 8c shows the trapping forces *F*_*x*_ on the targeted yeast cells with different diameters as functions of the shift distance of FP 2. It can be seen that although *F*_*x*_ undergoes a fluctuation at the beginning of the shift, which is due to the outputted optical field redistribution with the targeted cell shifted, *F*_*x*_ remains negative during the shift process, indicating that the yeast cell will keep trapped by FP 2. Therefore, the presented method can also be used for the regulation of cell chain consisted of spherical cells. Compared with the reported methods using multiple optical traps, the presented FPs approach has the advantages of easy fabrication, simple configuration, and high flexibility of manipulation. Moreover, the FPs can be precisely adjusted to manipulate the specific cells at different depths in the cell suspension. It should be pointed out that although the formation control of cells in this work is limited to a chain, more types of cell patterns are expected to be formed and regulated by employing multiple fiber arrays with the presented method.

In conclusion, an optical method has been demonstrated to realize the regulation of cell chain by using two FPs. Targeted *E. coli* cells were trapped by the FP and removed from the cell chain. The *E. coli* cells were then added back to the chain at different positions to change the cell contact sequence. Numerical simulations interpret the regulation process and indicate that the method can be used for the regulation of cell chains consisted of cell with different sizes and shapes (*e.g*. spherical). By incorporating the FPs into lap-on-chip platforms, the presented regulation method is expected to enable a new opportunity for the investigation of the cell growth, intercellular singling pathway, and pathogenic processes.

## Methods

### Fabrication of FPs

The fiber probes were fabricated through a flame-heating technique with the commercial single-mode optical fibers (connector type: FC/PC, core diameter: 9 μm, cladding diameter: 125 μm; Corning Inc.). Firstly, the buffer and polymer jacket were stripped off with a fiber stripper, and then the fibers were sheathed with a glass capillary (inner diameter: ~0.9 mm, wall thickness: ~0.1 mm, length: ~120 mm) to protect the fibers from breakage and warping. Secondly, the fibers were heated for approximately 1 min to reach their melting point. Then the drawing speed of about 0.5 and 0.6 mm/s were applied to the heated fibers 1 and 2, respectively. Meanwhile, the fiber diameters were decreased from 125 μm to 5.5 and 4 μm over a 2-mm and 2.5-mm length, respectively. Finally, the drawing speeds were increased to about 2 and 3 mm/s for fibers 1 and 2, respectively. Then the fibers were broken and FPs were fabricated with different abrupt tapered tips.

### Suspension preparation

The *E. coli* (DH5α) bacteria were grown at 37 °C in Lysogeny broth (LB) and then washed in phosphate buffered saline (PBS) buffer. To make sure the bacteria in a high-active condition, LB was added into the suspension and the bacteria in the logarithmic phase were chosen. After resuspended in the PBS buffer (diluted with deionized water) to desired concentrations (bacteria density: ~1 × 10^5^ #/mL), the suspension was dropped with a pipette onto the surface of a glass slide.

## Additional Information

**How to cite this article**: Liu, X. *et al*. Optical regulation of cell chain. *Sci. Rep*. **5**, 11578; doi: 10.1038/srep11578 (2015).

## Supplementary Material

Supplementary Information

Supplementary Video S1

Supplementary Video S2

Supplementary Video S3
