# Supplementary material for: Optical regulation of cell chain
Source: Sci Rep. 2015 Jun 22;5:11578. doi: 10.1038/srep11578 (PMC4476432; doi:10.1038/srep11578)
Supplement: Supplementary Information [file srep11578-s1.doc]

Supporting Information

Optical regulation of cell chain

*Xiaoshuai Liu, Jianbin Huang, Yao Zhang^*^, and Baojun Li^*^*

*State Key Laboratory of Optoelectronic Materials and Technologies, School of Physics and Engineering, Sun Yat-Sen University, Guangzhou 510275, China.*

*Corresponding authors: [zhyao5@mail.sysu.edu.cn](mailto:zhyao5@mail.sysu.edu.cn) (Y. Z.); [stslbj@](mailto:stslbj@mail.sysu.edu.cn)outlook.com (B. L.).

**1. Removal of *E. coil* 2 from the cell chain**

After forming a cell chain consisted of nine *E. colis* by using FP 1, FP 2 was manipulated by adjusting the microstage 2 to approach *E. coil* 2 at *t* = 0 s (Fig. S1a). After the 980-nm laser beam (*P*: 50 mW) was injected into FP 2 at *t* = 1 s, *E. coil* 2 started to be rotated and was finally orientated along the axial direction of FP 2 (Fig. S1b−e). Then *E. coil* 2 was removed from the cell chain with the shift of FP 2 along −*x* direction (Fig. S1f).


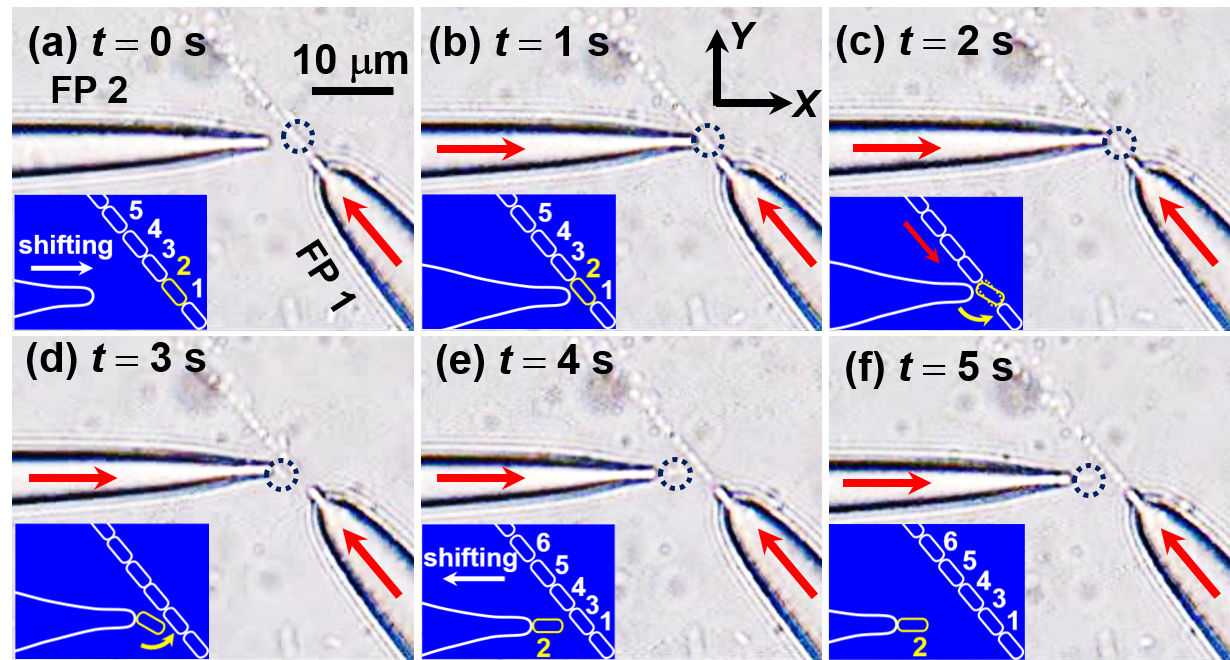


Fig. S1 Optical microscopic images for removing *E. coli* 2 from the cell chain consisted of nine *E. colis*. The insets schematically show the removal process. (a) FP 2 was adjusted to approach *E. coil* 2 of the cell chain. (b−e) Turing on the laser in FP 2, *E. coli* 2 was rotated and then gradually orientated along the axial direction of FP 2. (f) *E. coli* 2 was removed from the cell chain and then shifted with FP 2.

**2. Exchanging tips of a targeted cell contacted with neighboring cells**

After forming a cell chain consisted of six *E. colis* by using FP 1, FP 2 was manipulated by adjusting the microstage 2 to approach *E. coil* 3 at *t* = 0 s (Fig. S2a). Note that in this process, FP 2 was adjusted to approach the downside of *E. coil* 3 to ensure the negative torque (yielded by FP 2) exerted on the cell. After injecting the 980-nm laser into FP 2 at *t* = 1 s (Fig. S2b), *E. coil* 3 started to be rotated clockwise and gradually orientated along FP 2 due to the negative optical torque (Fig. S2c). Then by turning off the laser in FP 2, *E. coil* 3 was rotated clockwise (Fig. S2d) and finally oriented along the axial direction of FP 1 (Fig. S2e,f). The total rotation angle of *E. coil* 3 in this process was 180°, *i.e.*, the cell was inversed in the cell chain. Therefore, the tips of *E. coil* 3 contacted with *E. colis* 2 and 4, indicated by yellow and red dots in Fig. S2, were exchanged.


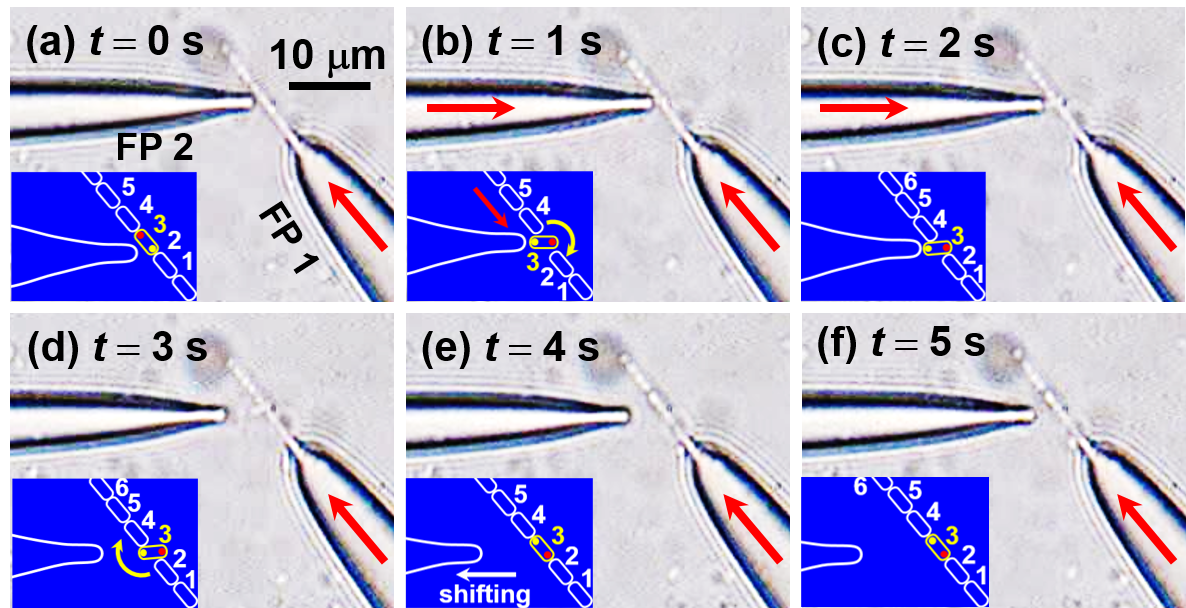


Fig. S2 Optical microscopic images for the rotation of *E. coil* 3 in the cell chain consisted of six *E. colis*. (a) FP 2 was adjusted to approach the downside tip (indicated by the yellow dot) of *E. coil* 3 in the cell chain. (b,c) After turning on the laser in FP 2, *E. coil* 3 was rotated counterclockwise and then trapped by FP 2. (d,e) After turning off the laser in FP 2, *E. coil* 3 was gradually orientated along the axial direction of FP 1 again. (f) *E. coil* 3 was inversed in the cell chain and tips of *E. coil* 3 contacted with *E. colis* 2 and 4 were exchanged.
